# Triggering Apoptotic Death of Human Malignant Melanoma A375.S2 Cells by Bufalin: Involvement of Caspase Cascade-Dependent and Independent Mitochondrial Signaling Pathways

**DOI:** 10.1155/2012/591241

**Published:** 2012-04-07

**Authors:** Yu-Ping Hsiao, Chun-Shu Yu, Chien-Chih Yu, Jai-Sing Yang, Jo-Hua Chiang, Chi-Cheng Lu, Hui-Ying Huang, Nou-Ying Tang, Jen-Hung Yang, An-Cheng Huang, Jing-Gung Chung

**Affiliations:** ^1^Institute of Medicine, Chung Shan Medical University, Taichung 402, Taiwan; ^2^Department of Dermatology, Chung Shan Medical University Hospital, Taichung 402, Taiwan; ^3^Department of Dermatology, Jen-Ai Hospital, Taichung 412, Taiwan; ^4^School of Pharmacy, China Medical University, Taichung 404, Taiwan; ^5^Department of Pharmacology, China Medical University, Taichung 404, Taiwan; ^6^Department of Life Sciences, National Chung Hsing University, Taichung 402, Taiwan; ^7^Department of Nutrition, China Medical University, Taichung 404, Taiwan; ^8^School of Chinese Medicine, China Medical University, Taichung 404, Taiwan; ^9^School of Medicine, Tzu Chi University, Hualien 970, Taiwan; ^10^Department of Dermatology, Buddhist Tzu Chi General Hospital, Hualien 970, Taiwan; ^11^Department of Nursing, St. Mary's Medicine Nursing and Management College, Yilan 266, Taiwan; ^12^Department of Biological Science and Technology, China Medical University, Taichung 404, Taiwan; ^13^Department of Biotechnology, Asia University, Taichung 413, Taiwan

## Abstract

Bufalin was obtained from the skin and parotid venom glands of toad and has been shown to induce cytotoxic effects in various types of cancer cell lines, but there is no report to show that whether bufalin affects human skin cancer cells. The aim of this investigation was to study the effects of bufalin on human malignant melanoma A375.S2 cells and to elucidate possible mechanisms involved in induction of apoptosis. A375.S2 cells were treated with different concentrations of bufalin for a specific time period and investigated for effects on apoptotic analyses. Our results indicated that cells after exposure to bufalin significantly decreased cell viability, and induced cell morphological changes and chromatin condensation in a concentration-dependent manner. Flow cytometric assays indicated that bufalin promoted ROS productions, loss of mitochondrial membrane potential (ΔΨ_*m*_), intracellular Ca^2+^ release, and nitric oxide (NO) formations in A375.S2 cells. Additionally, the apoptotic induction of bufalin on A375.S2 cells resulted from mitochondrial dysfunction-related responses (disruption of the ΔΨ_*m*_ and releases of cytochrome *c*, AIF, and Endo G), and activations of caspase-3, caspase-8 and caspase-9 expressions. Based on those observations, we suggest that bufalin-triggered apoptosis in A375.S2 cells is correlated with extrinsic- and mitochondria-mediated multiple signal pathways.

## 1. Introduction

Skin cancer is one of the major causes of cancer death worldwide [1, 2]. Human cutaneous malignant melanoma is an aggressive skin cancer, and its incidence still continues to rise in individuals of European origin worldwide [3]. About 4% of all dermatologic cancers are melanoma, but it is responsible for more than 80% of deaths from skin cancers, and patients with metastatic melanoma have a 10-year survival rate that is less than 10% [4, 5]. It is recognized that melanoma is highly resistant to conventional chemotherapy, which has preferential metastasis to the brain, lung, liver, and skin [6, 7]. Currently, the effective cure rate of melanoma has not been achieved with surgery, radiation, or chemotherapy.

For inhibiting the development of cancer, chemoprevention and chemotherapy are commonly used, and those agents belong to pharmacological or natural agents [8–10]. Chemoprevention has been shown to prevent a wide variety of cancers in multiple animal models [11]. Natural agents include antioxidants and cancer preventative agents or even act as cancer therapy drugs [12, 13].

Bufalin, one of the major digoxin-like components, is a bufadienolide purified from Chan-Su extracts from the venom of *Bufo bufo gargarizan *[14, 15], and it has long been used as a treatment for heart failure in Chinese medicine in Asian countries [16, 17]. Bufalin acts as a Na^+^-K^+^-ATPase inhibitor for increasing the intracellular Ca^2+^ concentration [14, 18] and as a topoisomerase II inhibitor [19, 20]. Bufalin induced leukemia cell differentiation and apoptotic death in prostate cancer cells [21–23], and it inhibited hepatocellular carcinoma solid tumor growth *in vivo* [24]. Recently, the reports have shown that bufalin inhibited cell proliferation of human lung cancer cells [25] and induced apoptosis of hepatoma Hep G2 cells [26]. In our laboratory, we also found that bufalin suppressed the migration and invasion of human osteosarcoma U-2 OS cells by suppression of the matrix metalloproteinase-2 (MMP-2), extracellular signal-regulated kinase (ERK), and c-Jun N-terminal kinase (JNK) signaling pathways [27]. However, there is no report regarding bufalin-induced apoptotic death in human malignant skin cancer cells *in vitro*. The present study investigated bufalin-triggered cell death in human malignant melanoma A375.S2 cells *in vitro*. Due to our observations, the induction of apoptotic death in A375.S2 cells by bufalin is mediated through caspase cascade-dependent and independent mitochondrial signaling pathways.

## 2. Materials and Methods

### 2.1. Chemicals and Reagents

Bufalin, dimethyl sulfoxide (DMSO), propidium iodide (PI), RNase A, and Triton X-100 were obtained from the Sigma-Aldrich Corp. (St. Louis, MO, USA). Minimum essential media (MEM), penicillin-streptomycin, trypsin-EDTA, fetal bovine serum (FBS) and the L-glutamine were obtained from Gibco/Life Technologies (Carlsbad, CA, USA). 4,6-diamidino-2-phenylindole dihydrochloride (DAPI), 2′,7′-dichlorfluorescein-diacetate (DCFH-DA), 3′,3-dihexyloxacarbocyanine iodide (DiOC_6_), Fluo-3/AM, and 4-amino-5-methylamino-2′,7′-difluorofluorescein diacetate (DAF-FM) were obtained from the Molecular Probes/Life Technologies (Eugene, OR, USA). Sources of antibodies used in this study were as follows. Polyclonal antibodies specific for caspase-3, caspase-8, and caspase-9 were obtained from the Cell Signaling Technology Inc. (Danvers, MA, USA). Monoclonal antibodies specific for cytochrome *c*, apoptosis-inducing factors (AIF), Bcl-X, Fas, Fas ligand (FasL), glucose-regulated protein 78 (GRP78), *β*-actin, and horseradish peroxidase- (HRP-) conjugated secondary antibodies were obtained from the Santa Cruz Biotechnology, Inc. (Santa Cruz, CA, USA). Anti-endonuclease G (Endo G) (Cat. AB3639) and anti-Bax (Cat. 04-434) were bought from Merck Millipore (Billerica,MA,USA).

### 2.2. Cell Culture

The human malignant melanoma cell line (A375.S2) was purchased from the Food Industry Research and Development Institute (Hsinchu, Taiwan). About 1 × 10^6^ cells/mL were maintained in 75 cm^2^ tissue culture flasks with 90% MEM supplemented with 10% FBS, penicillin-streptomycin (100 U/mL penicillin and 100 *μ*g/mL streptomycin), and 2 mM L-glutamine and grown at 37°C in 100% humidity, 5% CO_2_, and 95% air [7, 28].

### 2.3. Assessment of Cell Morphological Changes and Viability

A375.S2 cells at the density of 2 × 10^5^ cells/well were placed onto 12-well plates and incubated at 37°C for 24 h before being treated with or without various concentrations (18.75, 37.5, 75, 150, 250, 350, and 450 nM) of bufalin for 24 or 48 h. About 0.5% DMSO (solvent) was used for the vehicle control regimen. At the end of incubation, cells were examined and photographed under contrast phase microscope for morphological changes determination as described elsewhere [29]. Then cells (1 × 10^5^ cells per sample) were centrifuged at 1000 ×g for 5 min, and cell pellets were resuspended with 0.5 mL of PBS containing 5 *μ*g/mL PI. The viable cells were determined by a PI-exclusion method and using a FACSCalibur flow cytometer (BD Biosciences, San Jose, CA, USA) for determination of viable cells as previously described [27, 30].

### 2.4. DAPI Staining for Apoptotic Cells

A375.S2 cells at a density of 2 × 10^5^ cells/well were placed onto 6-well plates and treated with bufalin (0, 150, 250, 350, 450, and 550 nM) for 24 and 48 h before cells from each treatment were isolated for DAPI staining as described previously [31, 32]. After staining, the cells were examined and photographed using a fluorescence microscope at 200x magnification [33, 34].

### 2.5. Determinations of the Reactive Oxygen Species (ROS) Production, the Levels of Mitochondrial Membrane Potential (ΔΨ_*m*_), Intracellular Ca^2+^ Release, and Nitric Oxide (NO) Generation

A375.S2 cells at a density of 2 × 10^5^ cells/well onto 12-well plates were exposed to 450 nM bufalin for 0, 1, 3, 6, 12, or 24 h to determine the changes in the levels of ROS, ΔΨ_*m*_, intracellular Ca^2+^ release, and NO formation, respectively. Cells were harvested from each treatment then resuspended in 500 *μ*L of DCFH-DA (10 *μ*M) for ROS (H_2_O_2_) determination, DiOC_6_ (1 *μ*mol/L) for ΔΨ_*m*_, Fluo-3/AM (2.5 *μ*g/mL) for intracellular Ca^2+^ concentrations, and DAF-FM (a nitric oxide indicator) for NO assessment and incubated at 37°C for 30 min and then were analyzed by flow cytometry [29, 34, 35].

### 2.6. Apoptotic Death-Associated Protein Levels Were Examined by Western Blotting

A375.S2 cells at a density of 1 × 10^6^ A375.S2 cells in 6-well plates were treated with 450 nM bufalin for 0, 6, 12, 24, and 48 h. Cells were harvested from each treatment by centrifugation for the total protein determination as PRO-PREP protein extraction solution (iNtRON Biotechnology, Seongnam-si, Gyeonggi-do, Korea) for Western blotting. The protein levels of caspase-3, caspase-8, caspase-9, cytochrome *c*, AIF, Endo G, Bcl-X, Bax, Fas, FasL, and GRP78 were examined by using sodium dodecylsulfate polyacrylamide gel electrophoresis (SDS-PAGE) as described previously [34–36]. After electrophoresis, the proteins were transferred to the Immobilon-P transfer membrane (Cat. IPVH00010, Merck Millipore) as described elsewhere [30, 33]. The appropriate horseradish peroxidase- (HRP-) conjugated secondary antibodies was used to visualize by Immobilon Western Chemiluminescent HRP substrate (Millipore) and X-ray film (GE Healthcare, Piscataway, NJ, USA). The density of bands was quantified using ImageJ 1.45 program [30].

### 2.7. Statistical Analysis

The statistical differences between the bufalin-treated and control samples were calculated by Student's *t*-test. A value of *P < 0.05 was considered significant. The quantitative data are shown as mean ± SD. The results from the *in vitro* studies are representative of at least two or three independent experiments.

## 3. Results

### 3.1. Bufalin Affects Cell Morphological Changes and Reduces Percentage of Cell Death in A375.S2 Cells

**Figure 1:**
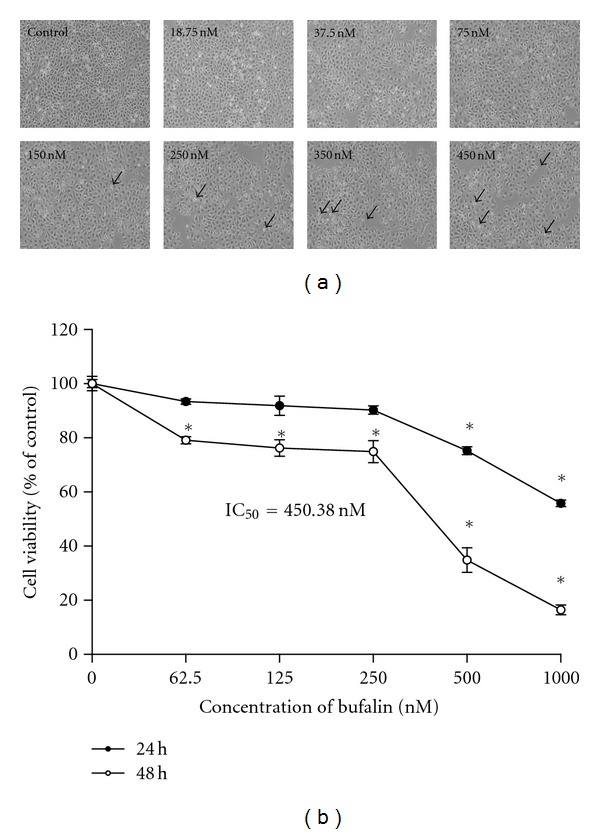
Bufalin affects cells' morphological changes and reduces percentage of viable A375.S2 cells. Cells were plated onto MEM + 10% FBS with various concentrations of bufalin for 24 or 48 h. (a) The morphological changes were examined and photographed under phase contrast microscope, and (b) the total percentages of viable cells were determined utilizing a PI-exclusion method and by flow cytometry as described in Section 2. Each point is mean ± SD of three experiments, *P < 0.05 was significantly different from the untreated control.

In order to investigate the biological effects of bufalin, A375.S2 cells were treated with various concentrations of bufalin for 24 and 48 h, and cell morphological changes and cell death were determined. The results are shown in Figures 1(a) and 1(b), which indicated that bufalin induced morphological changes (Figure 1(a)) and caused cell death in a concentration-dependent manner (Figure 1(b)). We found that the half maximal inhibitory concentration (IC_50_) is 450.38 nM in bufalin-treated A375.S2 cells at a 48 h incubation. Based on these observations, we selected the concentration of 450 nM bufalin, which is close to IC_50_, for further assessing whether the growth-inhibitory and cell death effects of bufalin are accompanied by its effect on apoptotic cell death.

### 3.2. Bufalin Triggers Apoptotic Cell Death in A375.S2 Cells

**Figure 2:**
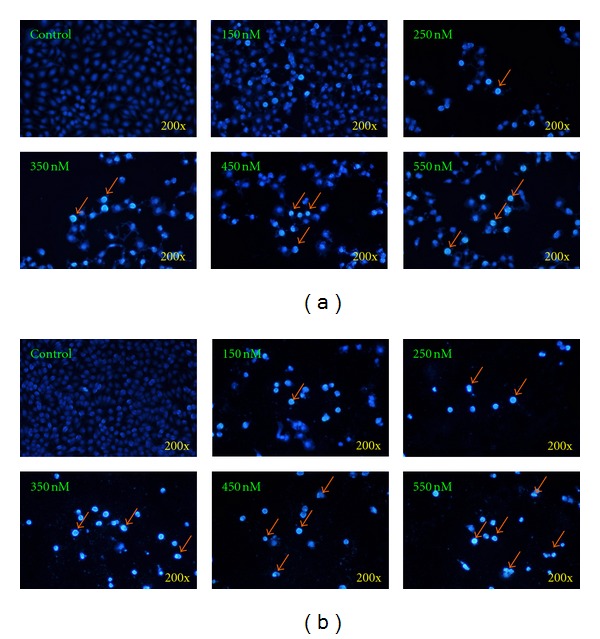
Bufalin induces chromatin condensation and apoptosis in A375.S2 cells. Cells were incubated with various concentrations of bufalin for 24 (a) and 48 h (b). Apoptotic cells were determined by DAPI staining and were photographed at a 200x magnification under fluorescence microscopy as described in Section 2. Each experiment was done with triple sets with similar results.

In order to confirm if bufalin-induced cell death in A375.S2 cells was through the induction of apoptosis, we used DAPI staining assay. Results shown in Figures 2(a) and 2(b) revealed that chromatin condensation and apoptotic cells were present in bufalin-treated A375.S2 cells for 24 and 48-h treatments. Moreover, the percentage of apoptotic cells is calculated (Figure 2) compared with intact control cells and this effect was concentration dependent.

### 3.3. Bufalin Affects the Levels of ROS, ΔΨ_*m*_, and Intracellular Ca^2+^ and NO in A375.S2 Cells

**Figure 3:**
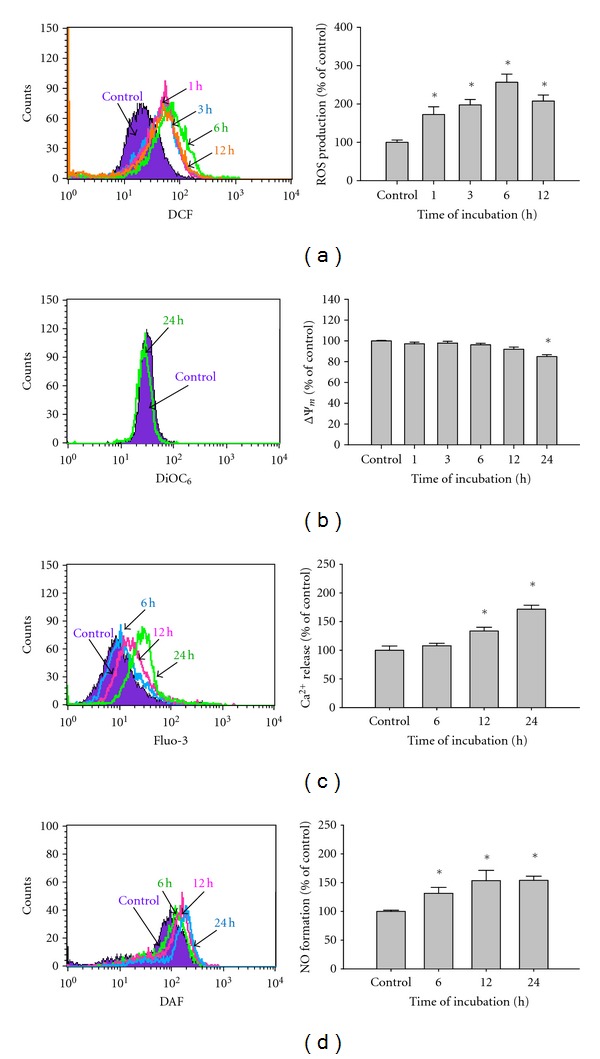
Bufalin influences the levels of reactive oxygen species (ROS), mitochondria membrane potential (ΔΨ_*m*_), intracellular Ca^2+^, and nitric oxide (NO) in A375.S2 cells. Cells were incubated with 450 nM bufalin for various time periods, before being stained by DCFH-DA for ROS level (a), by Fluo-3/AM for the intracellular Ca^2+^ level determined (b), and stained with DiOC_6_ for the ΔΨ_*m*_ levels (c), or cells were analyzed for NO production after being stained with DAF-FM (d) by flow cytometric analysis as described in Section 2. Each point is performed mean ±S.D. and was done in triplicate; *P < 0.05 was significantly different from the untreated control.

In order to further examine whether bufalin-induced apoptotic death is due to affecting the levels of ROS, ΔΨ_*m*_, intracellular Ca^2+^ and NO in A375.S2 cells. Cells were incubated with 450 nM bufalin for various time periods. The levels of ROS production, ΔΨ_*m*_, and Ca^2+^ and NO generation were measured by flow cytometric assay, and these results can be seen in Figure 3. The data demonstrated that bufalin promoted ROS (Figure 3(a)), intracellular Ca^2+^ production (Figure 3(c)), and NO formation (Figure 3(d)) in a time-dependent manner. We also found that bufalin decreased the levels of ΔΨ_*m*_ in A375.S2 cells at a 24 h exposure (Figure 3(b)).

### 3.4. Bufalin Alters in Apoptotic-Associated Proteins in A375.S2 Cells

**Figure 4:**
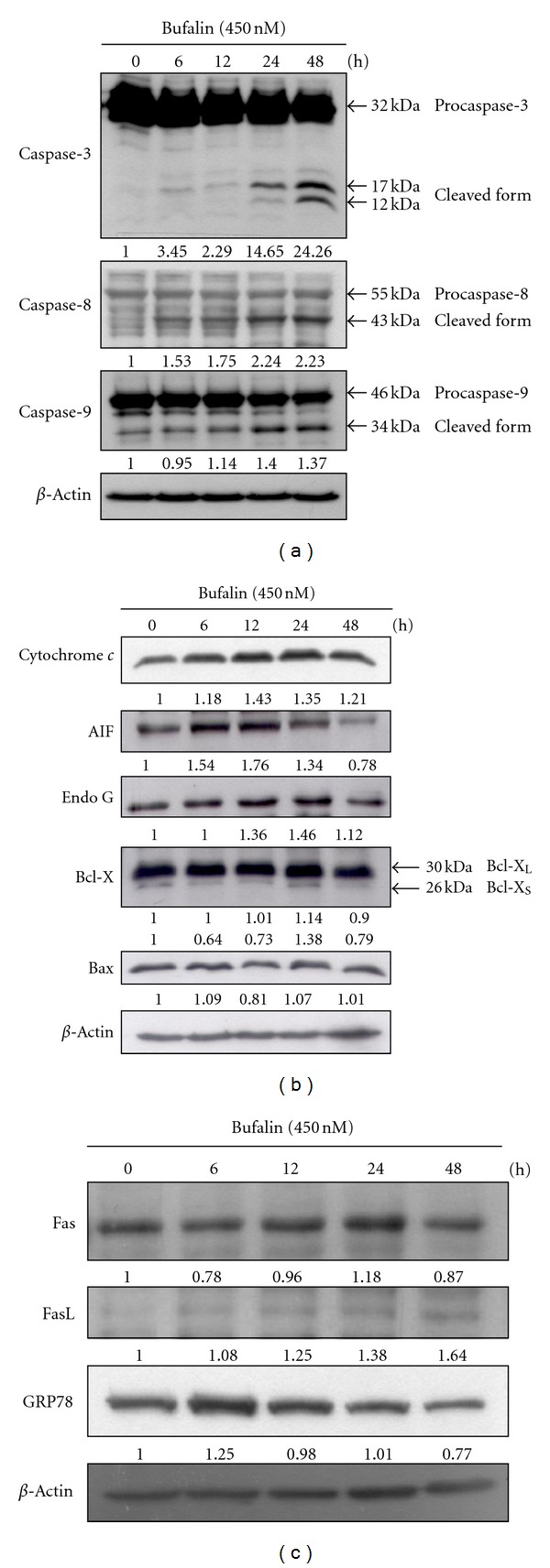
Bufalin alters the apoptosis-related protein levels in A375.S2 cells. A total of 1 × 10^6^ A375.S2 cells in 6-well plates were treated with 450 nM bufalin for 0, 6, 12, 24, and 48 h. Cells were harvested from each sample and associated proteins were measured by using SDS-PAGE and Western blotting as described in Section 2. The protein levels of caspase-3, caspase-8, and caspase-9 (a), cytochrome *c*, AIF, Endo G, Bcl-X, and Bax (b), and Fas, FasL, and GRP78 (c) expressions were examined. *β*-Actin was used as an internal control to ensure equal loading.

To further examine whether or not bufalin induces apoptosis *via* alterations of associated protein levels in A375.S2 cells, cells were treated with 450 nM bufalin for 0, 6, 12, 24, and 48 h and then were examined by Western blotting. The results shown in Figure 4 indicated that bufalin stimulated the expressions of cleaved caspase-3, cleaved caspase-8, cleaved caspase-9 (Figure 4(a)), cytochrome *c*, AIF, Endo G and Bax (Figure 4(b)) and Fas, FasL, and GRP78 (Figure 4(c)). However, bufalin decreased the expression of Bcl-X_L_ (Figure 3(b)) in A375.S2 cells.

## 4. Discussion

Several reports have shown that bufalin inhibited cell growth *via* cell cycle arrest and induction of apoptosis in many types of cancer cell lines [21–26]. However, there is no report to show that bufalin induced cell death in human melanoma A375.S2 cells. In the present study, we found that bufalin significantly induced cell morphological changes and decreased the percentage of viable A375.S2 cells (*P* < 0.05) at 24 and 48 h (Figures 1(a) and 1(b)). Furthermore, results from DAPI staining showed that bufalin induced apoptosis in A375.S2 cells in a concentration-dependent manner (Figures 2(a) and 2(b)). Although many experiments have showed that bufalin induced apoptosis in human cancer cell lines, the molecular mechanism of apoptotic induction in A375.S2 melanoma cells remains unclear. Thus, the present study further investigated the effect of bufalin on the intrinsic pathway of apoptosis in A375.S2 cells.

Apoptosis (programmed cell death type I) has been shown to perform *via* the death receptor-dependent pathway (extrinsic pathway) and mitochondria-dependent pathway (intrinsic pathway) [37–39]. In death receptor-dependent pathway, Fas and its receptor Fas ligand (FasL) and caspase-8 play an important role in regulating the induction of apoptosis in diverse cell types and tissues [40]. In the present study, the results from Western blotting (Figure 4(c)) indicated that bufalin promoted the expression of Fas and FasL and active form of caspase-8 (Figure 4(a)); these data demonstrated that bufalin induced apoptosis in A375.S2 cells through the death receptor-dependent pathway (extrinsic pathway). Figure 4(a) also shows that bufalin promoted the active form of caspase-3 in a time-dependent manner, which also indicated that bufalin induced apoptosis of A375.S2 cells *via* caspase-dependent pathway. It is well documented that caspase is normally present as an inactive procaspase but it can exist as a cleaved form when triggered [41, 42]. Those observations are in agreement with other reports which showed that bufalin induced apoptosis in human hepatocellular carcinoma cells and human prostate cancer cells *via* Fas and FasL pathway and caspase pathways [17, 26].

**Figure 5:**
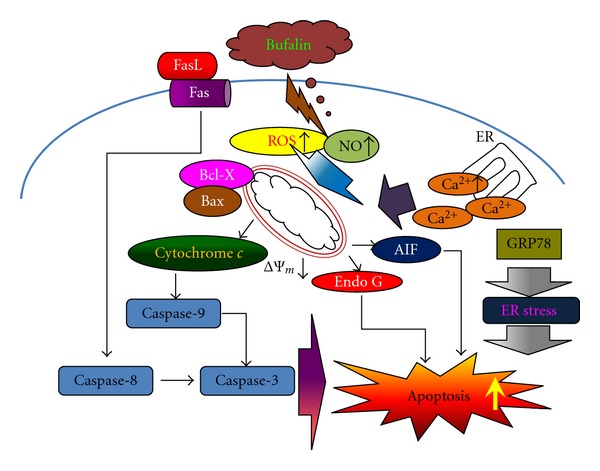
A schematic diagram showing the proposed signaling pathways of bufalin-induced apoptosis in A375.S2 human malignant melanoma cells.

Results from flow cytometric assay showed that bufalin decreased the levels of ΔΨ_*m*_ (Figure 3(b)). Furthermore, Figure 4(b) indicates that bufalin promoted the levels of cytochrome *c,* AIF, Endo G, and Bax but inhibited the Bcl-X_L_ expression in A375.S2 cells. Bcl-2 family proteins have been shown to regulate the mitochondria-dependent pathway and death receptor-dependent pathway [43, 44]. Bcl-2 family proteins include the proapoptotic proteins such as Bax, Bak, Bad, and Bcl-Xs and the antiapoptotic proteins such as Bcl-2, Bcl-X_L_, and Mcl-1 [45]. The ratio of Bax/Bcl-2 affects the levels of ΔΨ_*m*_ in cells after exposure to inducer of apoptosis [45], and then mitochondrial release of cytochrome *c* can be controlled by the ratio of Bax/Bcl-2 proteins and activated by proteolytic cleavage and heterodimerization [46]. Based on those observations, we suggest that bufalin-induced apoptosis in A375.S2 cells was partly through the mitochondria- as well as caspase-dependent and independent pathways. Overall, bufalin induced apoptosis in A375.S2 cells through cross-talk between the extrinsic and the intrinsic pathways. In conclusion, we proposed the possible signaling pathway of bufalin-induced apoptosis in A375.S2 cells that is shown in Figure 5. The possible flow chart indicated that bufalin triggers apoptosis *via* Fas/FasL, caspase cascade (caspase-3, 8 and 9) or the loss of ΔΨ_*m*_ in mitochondria and then led to AIF and Endo G release that is a novel finding for bufalin-induced apoptosis in A375.S2 cells. These results provided a novel and more detailed molecular mechanism in bufalin-induced apoptosis in A375.S2 cells* in vitro*.
